# Converse Flexoelectricity in van der Waals (vdW) Three-Dimensional Topological Insulator Nanoflakes

**DOI:** 10.1021/acs.jpcc.4c05690

**Published:** 2024-09-12

**Authors:** Qiong Liu, Srivilliputtur Subbiah Nanthakumar, Bin Li, Teresa Cheng, Florian Bittner, Chenxi Ma, Fei Ding, Lei Zheng, Bernhard Roth, Xiaoying Zhuang

**Affiliations:** † Faculty of Mathematics and Physics, Leibniz University Hannover, Hannover 30167, Germany; ‡ Department of Geotechnical Engineering, College of Civil Engineering, Tongji University, Shanghai 200092, China; § Institute of Plastics and Circular Economy (IKK), Faculty of Mechanical Engineering, Leibniz University Hannover, Hannover 30823, Germany; ∥ Institute of Solid State Physics, Faculty of Mathematics and Physics, Leibniz University Hannover, Hannover 30167, Germany; ⊥ Hannover Centre for Optical Technologies, Leibniz University Hannover, Hannover 30167, Germany; # Cluster of Excellence PhoenixD (Photonics, Optics and Engineering − Innovation Across Disciplines), Hannover 30167, Germany

## Abstract

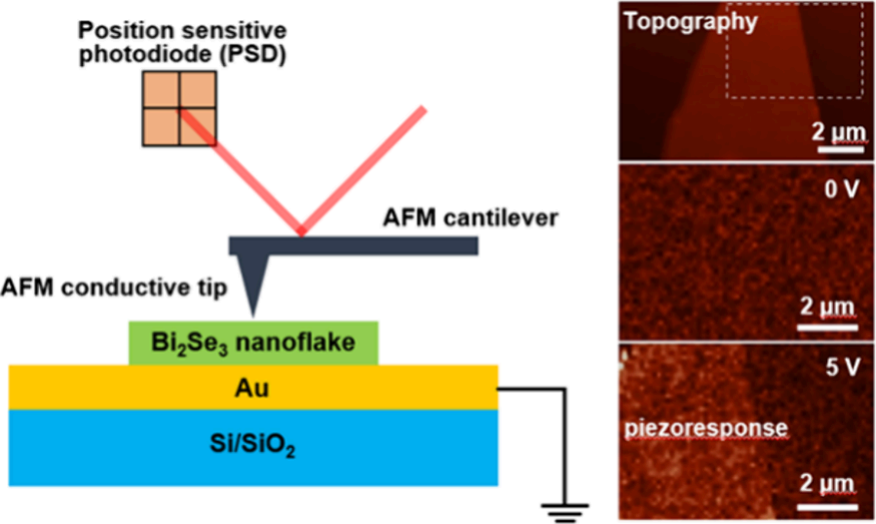

Low-dimensional van der Waals (vdW) three-dimensional (3D) topological insulators (TIs) have been overlooked, regarding their electromechanical properties. In this study, we experimentally investigate the electromechanical coupling of low-dimensional 3D TIs with a centrosymmetric crystal structure, where a binary compound, bismuth selenide (Bi_2_Se_3_), is taken as an example. Piezoresponse force microscopy (PFM) results of Bi_2_Se_3_ nanoflakes show that the material exhibits both out-of-plane and in-plane electromechanical responses. With careful analyses, the electromechanical responses are verified to arise from the converse flexoelectricity. The Bi_2_Se_3_ nanoflakes have a decreasing effective out-of-plane piezoelectric coefficient *d*_33_^eff^ with the thickness increasing, with the *d*_33_^eff^ value of ∼0.65 pm V^–1^ for the 37 nm-thick sample. The measured effective out-of-plane piezoelectric coefficient is mainly contributed by the flexoelectric coefficient, μ_39_, which is estimated to be approximately 0.13 nC m^–1^. The results can help to understand the flexoelectricity of low-dimensional vdW TIs with centrosymmetric crystal structures, which is crucial for the design of nanoelectromechanical devices and spintronics built by vdW TIs.

## Introduction

Two-dimensional (2D) van der Waals (vdW) materials are attracting great attention in various applications, owing to their novel mechanical, electrical, magnetic, and optical properties.^1−4^ Especially, 2D vdW materials are extremely thin, leading to considerable flexibility, which makes them ideal platforms to study electromechanical couplings, including piezoelectricity and/or flexoelectricity. The electromechanical properties of 2D vdW materials drive them in the arising next-generation devices, such as strain-related sensors and energy harvesters.^5,6^

In past years, computational methods have been widely used to study the electromechanical properties of materials.^7^ At the micro/nano scale, an advanced technique to characterize the electromechanical properties of low-dimensional materials is piezoresponse force microscopy (PFM).^8,9^ PFM can detect the electromechanical deformation caused by an out-of-plane electric field that is applied by an electrically conducting tip in atomic force microscopy (AFM). The applied electric field induces a strain, which is termed the converse piezoelectric effect. Another type of electromechanical coupling effect in PFM is converse flexoelectricity, which refers to the appearance of a strain induced by a polarization gradient. Converse flexoelectricity is the reverse phenomenon of flexoelectricity that describes the appearance of polarization arising from a strain gradient. Unlike piezoelectricity, flexoelectricity or converse flexoelectricity exists in both centrosymmetric and asymmetric dielectric materials.^8,9^

Topological insulators (TIs) are a material family that owns metallic conductivity on their boundaries due to the existence of electronic edge (in 2D TIs) or surface states (in three-dimensional (3D) TIs) while exhibiting bulk states with energy band gaps.^9,10^ Novel phenomena like quantum spin Hall effects and spin momentum locking pave the way for TIs to be building blocks in quantum computing and spintronics.^10−12^ Revealing the electromechanical properties of TIs can help in exploring further applications. For instance, TI piezotronics have been proposed based on the mechanism that piezoelectricity can modulate the electron transport in quantum wells.^13^ Investigations of electromechanical properties of 2D vdW materials take much interest in graphene,^14^ boron nitride (BN),^15^ and transition-metal dichalcogenides (TMDCs),^16^ and also others, among which graphene is known as a 2D TI.^17^ However, less attention was paid to revealing the electromechanical properties, especially the flexoelectricity of other kinds of low-dimensional nanostructures of 3D vdW TIs. Bismuth selenide (Bi_2_Se_3_) is a typical 3D TI with a single Dirac cone, belonging to the V–VI group chalcogenide material family.^10^ It is a promising candidate for the realization of spintronic devices.^10^

From a microscopic view, flexoelectricity in crystalline dielectric materials is contributed by two parts: ionic and electronic. Stengel et al. found from first principles that the two contributions exist in several kinds of 2D materials, such as graphene, BN, and TMDC monolayers. They built a predictive continuum model to connect their findings with the PFM measurements of the converse flexoelectric effects of these 2D materials.^18^ As a crystalline vdW dielectric material, the Bi_2_Se_3_ nanoflakes are most likely to comply with the model. Inspired by the study on the flexoelectricity of 2D TMDCs using PFM, we adopted the PFM technique to investigate the electromechanical properties of thin rhombohedral Bi_2_Se_3_ nanoflakes in this work. It is found that the Bi_2_Se_3_ nanoflakes have both out-of-plane and in-plane electromechanical responses. In PFM measurements, it is difficult to isolate flexoelectricity from piezoelectricity. The Bi_2_Se_3_ nanoflakes have a centrosymmetric crystal structure, exhibiting no bulk piezoelectricity. The electromechanical responses in the Bi_2_Se_3_ nanoflakes, therefore, are verified to solely originate from the flexoelectricity. The results may motivate the development of flexotronic devices built by vdW TIs with centrosymmetric structures.

## Methods

### Sample Fabrication

High-quality (purity >99.995%) rhombohedral Bi_2_Se_3_ bulk crystals were purchased from HQ Graphene Inc., Netherlands. Low-dimensional Bi_2_Se_3_ flakes were prepared using a facile mechanical exfoliation method, which involves using scotch tape to exfoliate a bulk Bi_2_Se_3_ crystal repeatedly. Briefly, a bulk Bi_2_Se_3_ crystal was placed on a piece of scotch tape, and then another piece of tape was pressed against the crystal and peeled off. The process was repeated several times. Next, the tape with Bi_2_Se_3_ nanoflakes was pressed against a homemade polydimethylsiloxane (PDMS) stamp prepared on a polished Si substrate by spin-coating. After the Scotch brand tape was slowly peeled off, some thin Bi_2_Se_3_ flakes were left on the PDMS stamp. The PDMS stamp with Bi_2_Se_3_ nanoflakes was placed against an Au-coated Si/SiO_2_ substrate, gently pressed, and released. Finally, low-dimensional Bi_2_Se_3_ flakes were left on the Au-coated Si/SiO_2_ substrate.

### Characterization

The geometry of the AFM tip was characterized by scanning electron microscopy (SEM, PIONEER Two, RAITH, Germany). PFM and KPFM measurements were performed in a contact mode and a noncontact mode, respectively, on an NTEGRA Prima scanning probe microscope (NT-MDT Co., Ireland) in ambient conditions using commercial conductive Cr/Au-coated silicon probes with a spring constant of ∼2.8 N m^–1^ and a free resonance frequency of ∼75 kHz. AC-driven voltages with a frequency of 60 kHz were applied between the probe tip and the Au layer on the Si/SiO_2_ substrate for both vertical PFM (VPFM) and lateral PFM (LPFM) measurements, where the PFM response was measured by a lock-in amplification technique.

## Results and Discussion

**Figure 1:**
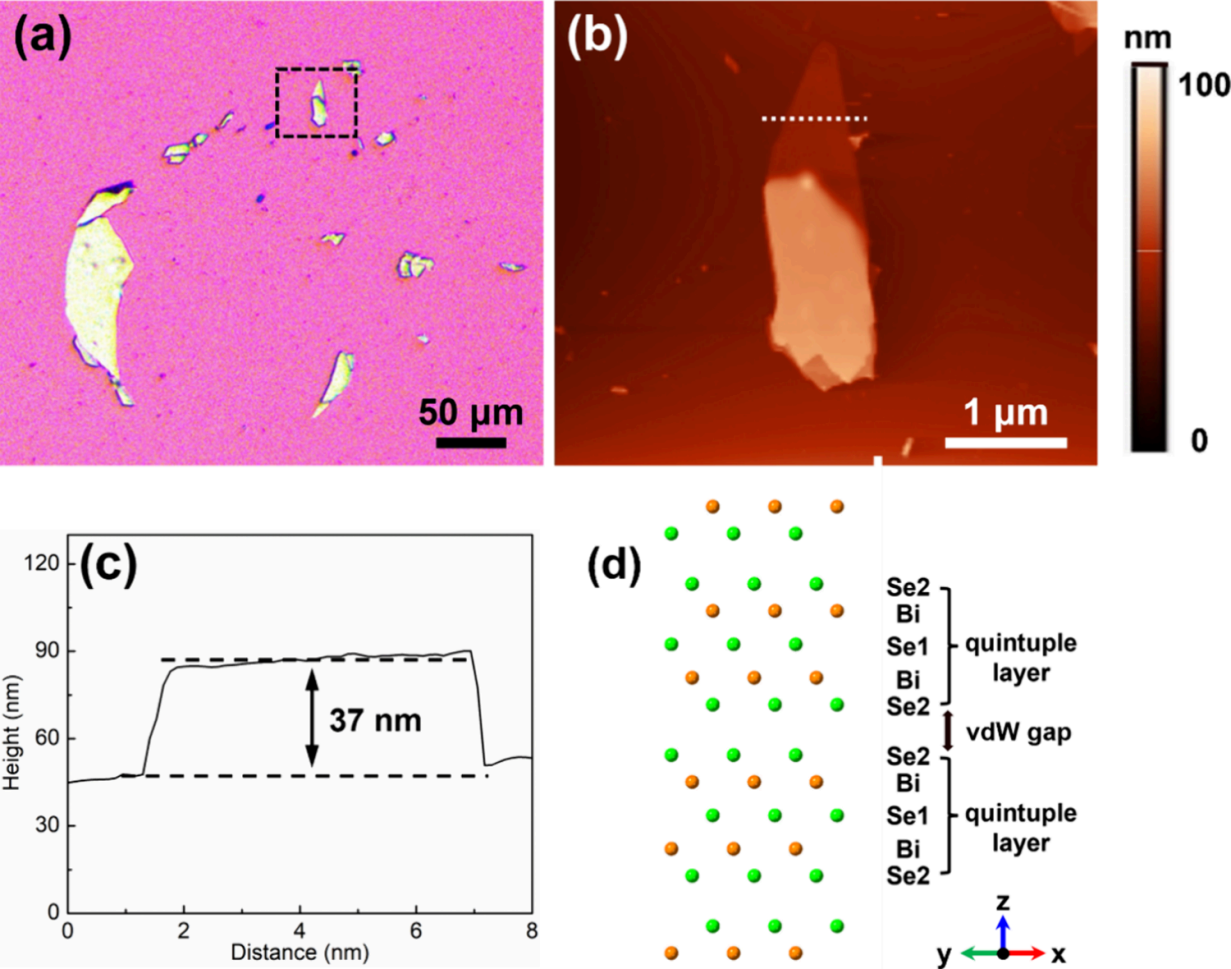
Sample characterization. (a) Optical microscopy image of the as-prepared Bi_2_Se_3_ flakes on a gold-coated Si/SiO_2_ substrate. (b) Contact-mode AFM topography image and (c) the corresponding height profile along the dashed line in (b) of the Bi_2_Se_3_ nanoflake in the framed region in (a). (d) Schematic of the crystal structure of Bi_2_Se_3_.

An optical microscope image of the as-obtained Bi_2_Se_3_ flakes is shown in Figure 1a. The topography of the thin Bi_2_Se_3_ nanoflakes situated on Au-coated Si/SiO_2_ substrates was obtained using contact-mode AFM (see Methods). Figure 1b shows a typical AFM image of a single Bi_2_Se_3_ nanoflake situated in the framed region in Figure 1a. The corresponding line section (Figure 1c) of the height shows that its thickness is 37 nm. The investigated Bi_2_Se_3_ has a rhombohedral crystal structure with a centrosymmetric space group (*R*3̅m), which belongs to D_3d_ point symmetry. Bulk Bi_2_Se_3_ can be considered to have a layered structure periodically stacked by the so-called quintuple layers with a vdW gap between every two quintuple layers, as shown in Figure 1d. A single quintuple layer consists of five atomic layers with the sequence −Se2–Bi–Se1–Bi–Se2–, where 1 and 2 denote two different chemical states for the Se anions, and the bond length between Bi and Se1 is smaller than that between Bi and Se2. The lattice parameters of Bi_2_Se_3_ are *a* = *b* = 0.413 nm, *c* = 2.856 nm, α = β = 90°, and γ = 120°. Since each unit cell spans over three quintuple layers, the thickness of a single quintuple layer is ∼0.955 nm.^19^ Thus, the thickness of the Bi_2_Se_3_ nanoflake in Figure 1b is approximately 39 layers.

PFM is often used to characterize piezoelectricity by applying an AC driving voltage (*V*_AC_) to the surface of the material via an electrically conductive AFM tip and detecting the piezoresponse signal. The experimental setup is schematically shown in Figure S1. The applied AC voltage induces an electric field between the AFM tip and the bottom Au electrode, resulting in expansion or contraction in the local volume of the material. The Au substrate can minimize the electrostatic effects. The displacements of the material cause oscillations of the AFM cantilever, which are detected by a position-sensitive photodiode (PSD) and read out with a lock-in amplifier. Since PFM can detect out-of-plane (vertical) and in-plane (lateral) surface displacements, both VPFM and LPFM techniques are employed to illuminate the electromechanical nature of Bi_2_Se_3_ nanoflakes. In PFM measurements, a lock-in amplifier can record the amplitude and phase signals while simultaneously acquiring the surface topography.

**Figure 2:**
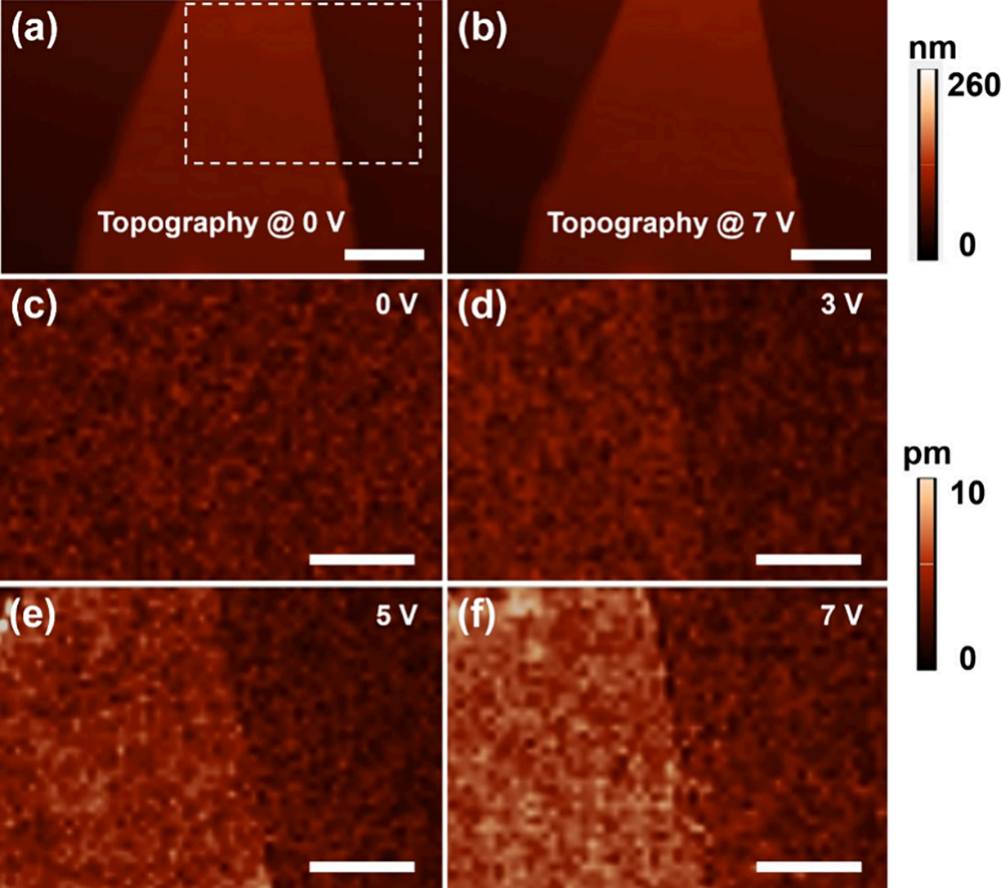
VPFM measurements of an individual Bi_2_Se_3_ nanoflake with a thickness of 37 nm. Contact-mode AFM images of the nanoflake under AC voltages of (a) 0 and (b) 7 V, respectively. VPFM amplitude images measured at AC voltages of (c) 0, (d) 3, (e) 5, and (f) 7 V, respectively. Scale bars: 2 μm.

Figure 2 shows the VPFM images under various AC voltages of 0, 3, 5, and 7 V, respectively. As shown in Figure 2a, no image contrast of 0 V can be observed, indicating no electromechanical responses. This suggests that without an external electric field, mechanical displacements cannot be induced. With *V*_AC_ increasing, the contrast comes out and becomes more remarkable, suggesting that the electromechanical coupling arises from Bi_2_Se_3_ and becomes stronger. Moreover, the electromechanical response signal should not be influenced by the crosstalk of the material topographic artifacts since the frequency (60 kHz) of the applied voltage is far from the contact resonance frequency (340 kHz),^16^ as shown in Figure S2. Even though there is no contrast in the amplitude image under an AC voltage of 0 V, the topography images acquired during PFM measurements almost remain unchanged (Figure 2a,b), which further confirms that the topographic artifacts can be ruled out.

**Figure 3:**
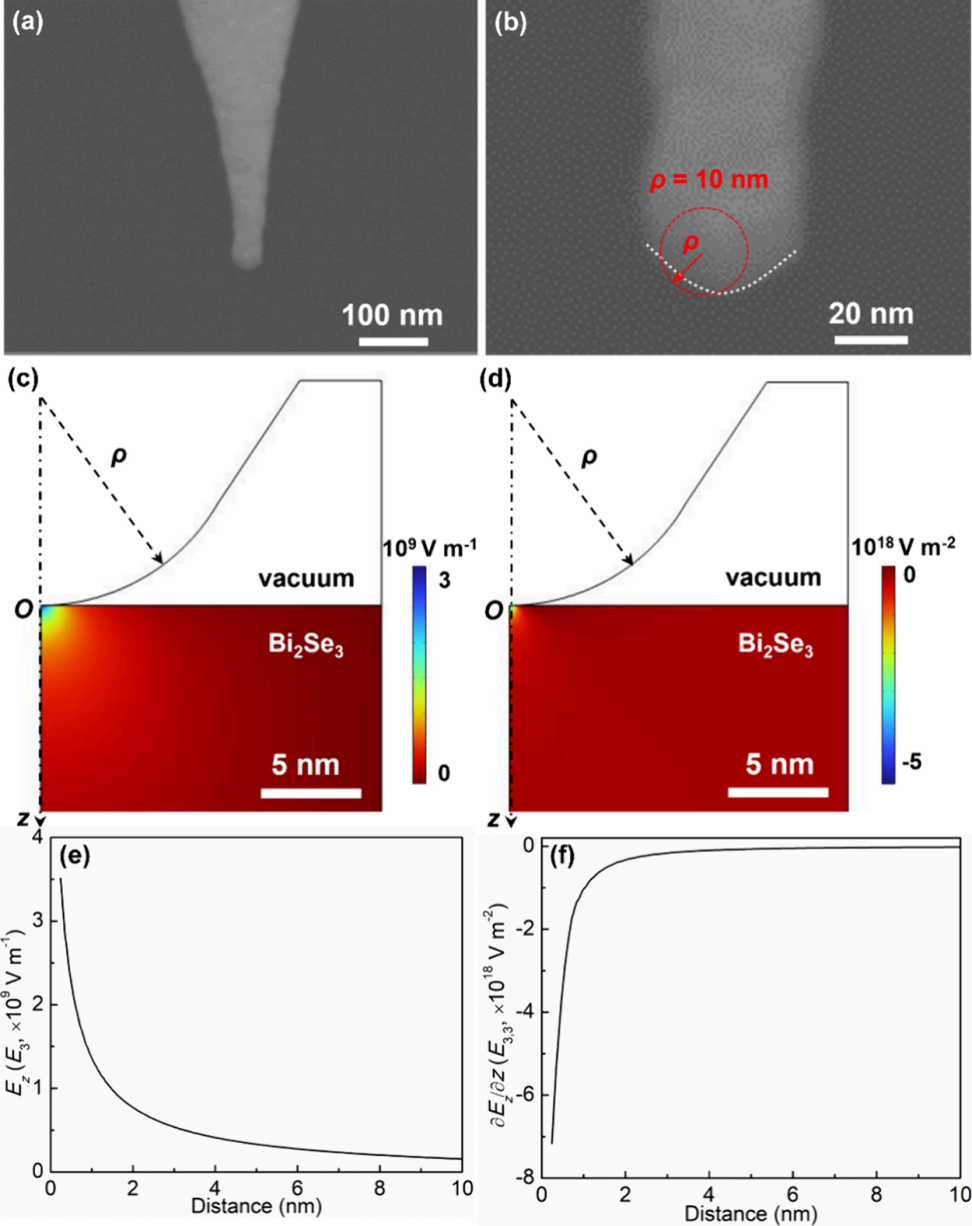
Geometry of the conductive AFM tip and contact of the tip with flat Bi_2_Se_3_. (a) SEM image of the used AFM conductive tip. ρ represents the radius of the tip head, which is 10 nm. (b) Zoomed-in SEM image of the framed area in panel a. (c, d) Models of distribution of the out-of-plane (along the *z* direction) electric field *E*_*z*_ (denoted as *E*_3_) and gradient (, denoted as *E*_3,3_) of *E*_*z*_ inside the Bi_2_Se_3_ plate below the tip apex, respectively, under an voltage of 8 V. The tip shape is set to be consistent with the one shown in (b). The thickness of the Bi_2_Se_3_ plate is 10 nm. (e, f) Line curves of *E*_3_ and *E*_3,3_ along the *z* direction.

Another possible contribution to the PFM signal amplitude could be from the electrostatic force between the tip-cantilever system and the sample, which is described as
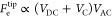
1where *F*_e_^tip^, *V*_DC_, *V*_C_, and *V*_AC_ represent the electrostatic force, the DC tip bias, the contact potential difference between the tip and the sample, and the AC tip bias, respectively.^20^ According to previous works, the interaction between the tip-cantilever and the sample can be suppressed by using a high aspect ratio tip as the one used in our experiments.^20^ As shown in Figure 3a, the tip used in this work has a cone shape with a round head whose radius is only ∼10 nm (Figure 3b). To confirm that the electrostatic force has a minimal impact on the total PFM signal, we performed the PFM tests on an individual Bi_2_Se_3_ nanoflake by applying different DC biases at a constant AC voltage of 8 V. As shown in Figure S3, the VPFM signal amplitude is independent of *V*_DC_, indicating that the electrostatic force plays a neglectable role.

In PFM measurements, the effective piezoresponse may not intrinsically originate from the piezoelectricity of the material, since an electric field gradient field is generated below the tip when a voltage is applied. The electric gradient can also generate a mechanical strain, which is called the converse flexoelectric effect.^21^Figure 3c–f shows the results of the simulated electrostatic field distribution. The dielectric constant of 100 for Bi_2_Se_3_ is adopted according to a previous work.^22^ The electric field distribution is obtained by solving the partial differential equations ∇ × ***D*** = *q* and ***E*** = −∇ φ using the finite element method in COMSOL Multiphysics, where ***D*** is the electric displacement and *q* is the surface charge of the AFP tip, which is taken as zero; ***E*** is the electric field and φ is the electric potential, which is set as a constant value along the AFM tip surface. Due to the cone shape of the AFM tip, an inhomogeneous electric field is generated beneath the tip (Figure 3c). According to Coulomb’s law, the electric field is most intense right below the tip apex and decays with increasing distance from the tip (Figure 3e), resulting in a radial pattern (Figure 3c).^8^ Simultaneously, the inhomogeneous electric field forms an electric field gradient field, which exhibits a pattern similar to that of the mother electric field, decaying from near the tip (Figure 3d,f). The electric field gradient field is likely to induce converse flexoelectric responses in PFM measurements. Thus, converse flexoelectricity and converse piezoelectricity always have a joint contribution to the piezoresponse in PFM measurements for piezoelectric materials. Due to the universal existence of flexoelectricity in all dielectric materials, a nonzero effective piezoelectric coefficient accounting for both piezoelectricity and flexoelectricity can be obtained in PFM tests.^8^ For Bi_2_Se_3_, the 3D TI properties can even be maintained at a very small thickness of three quintuple layers (∼2.88 nm).^23^ This indicates that even though surface gapless metallic states may exist at the top and bottom surfaces of the Bi_2_Se_3_ nanoflakes, the insulating bulk still acts as a dielectric layer between the AFM tip and the Au substrate. Therefore, effective piezoresponses can be detected for Bi_2_Se_3_ nanoflakes as common insulators and semiconductors in PFM measurements.

Converse piezoelectricity can be described as ε_*jk*_ = *d*_*ijk*_*E*_*k*_, where ε_*jk*_, *d*_*ijk*_, and *E*_*k*_ represent strain tensors, third-rank piezoelectric tensors, and electric fields, respectively; the subscripts *i*, *j*, and *k* ∈ {1, 2, 3}, and *jk* follow the Voigt notation. Thanks to the D_3d_ (*R*3̅m) crystal structure of the tested Bi_2_Se_3_, all of the out-of-plane piezoelectric coefficients in the material are zero while its flexoelectric tensor has nonzero coefficients in the out-of-plane direction. It is reasonable to confirm that the measured piezoresponse solely arises from the converse flexoelectricity, allowing us to quantitatively study the flexoelectricity of the Bi_2_Se_3_ nanoflakes, as discussed later. The out-of-plane electromechanical response is usually quantified by the effective piezoelectric coefficient *d*_33_^eff^, which is given by *d*_33_^eff^ = *A*_p_/*V*_AC_, where *A*_p_ is the deformation displacement. With the deflection sensitivity, *S*_d_, of the AFM cantilever measured, *A*_p_ can be calculated by converting the electromechanical current signal (AMP) into physical deflection following the equation, *A*_p_ = AMP × *S*_d_/Gain.^24^ Owing to the internal noise of the device, nonzero amplitude signals always exist and are even detected on the Au substrates with or without drive voltages. The electromechanical response is superimposed by this kind of nonzero background signal.^25^ Therefore, a background subtraction method (Figure S4) is used to calculate the true amplitude arising from the deformation in Bi_2_Se_3_ using both the PFM amplitude and phase channels.^16^ Under this method, the PFM image taken on the Au substrate with the drive voltage serves as the background. As can be seen in Figure S5, the phase signals from the substrate and the Bi_2_Se_3_ under each driven voltage show no obvious difference (<1°). Therefore, the real electromechanical response from Bi_2_Se_3_ can be directly determined by the difference between the amplitude values measured on Bi_2_Se_3_ and those of the substrate.

**Figure 4:**
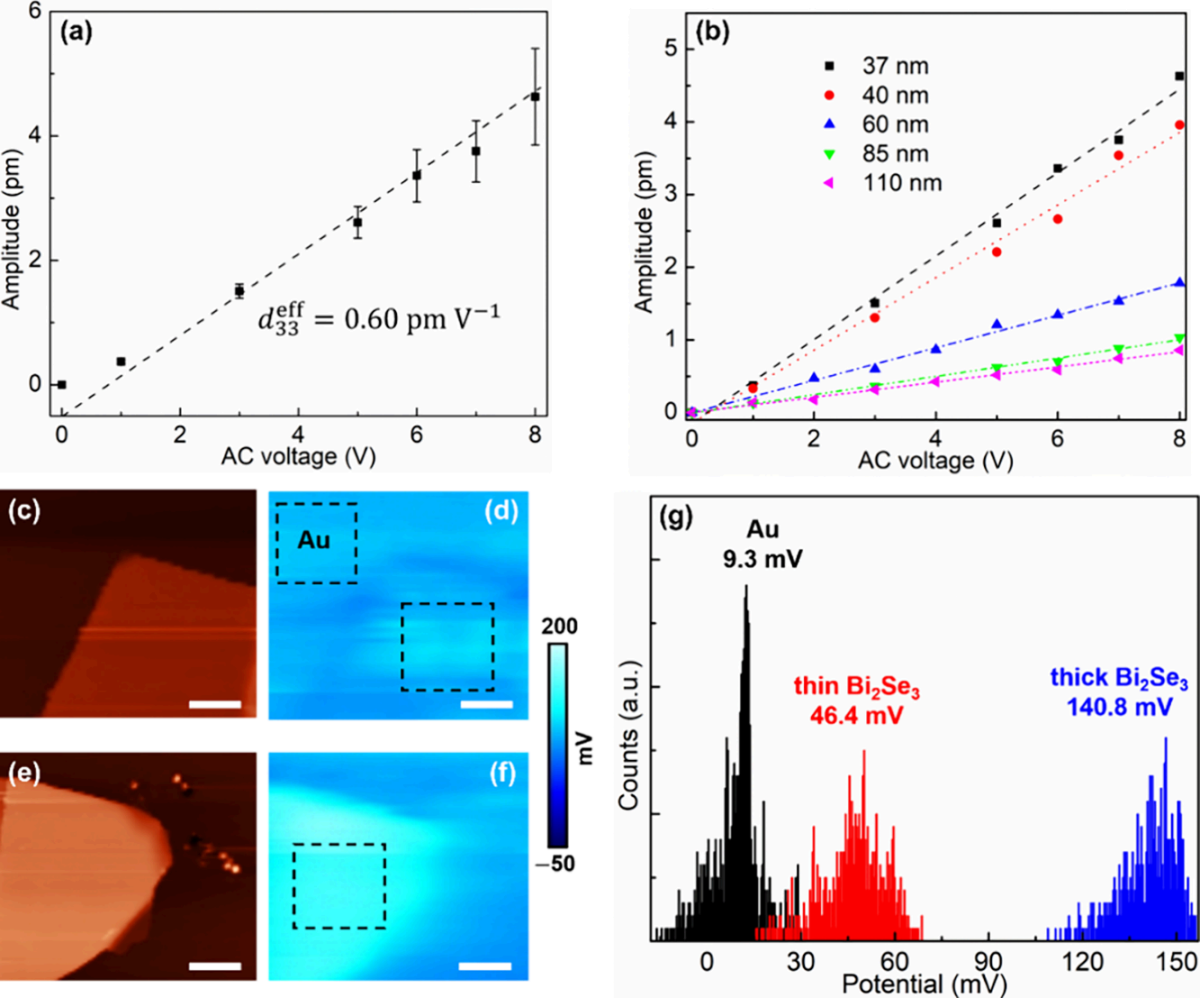
Out-of-plane electromechanical properties of Bi_2_Se_3_ nanoflakes. VPFM amplitude as a function of the applied AC voltage for (a) the nanoflake with a thickness of 37 nm and (b) nanoflakes with different thicknesses. (c) AFM topography image of a Bi_2_Se_3_ nanoflake with a thickness of 49 nm and (d) the corresponding KPFM image. (e) AFM topography image of a Bi_2_Se_3_ nanoflake with a thickness of 109 nm and (f) the corresponding KPFM image. Scale bars, 5 μm. (g) Statistical distribution of the surface potential of the framed regions in (d) and (f), corresponding to Au, the thin Bi_2_Se_3_ nanoflake and the thick Bi_2_Se_3_ nanoflake, respectively.

To more accurately calculate *d*_33_^eff^, the average amplitude value over a specific area for each drive voltage is adopted, which is obtained from the amplitude mapping of this area. Figure 4a shows the relationship between the deflection amplitude and the applied AC voltage. The deflection amplitude can be considered to linearly increase with the applied AC voltage, indicating that *d*_33_^eff^ is independent of the drive voltage. The value of *d*_33_^eff^ is then obtained from the slope of the fitting curve, which is 0.60 ± 0.04 pm V^–1^. This value is close to that (0.7 pm V^–1^) of InSe nanoflakes,^24^ but approximately one-third smaller than that (1.03 pm V^–1^) of the monolayer MoS_2_ reported by Yu et al.^16^ and is much smaller than those of some typical piezoelectric materials, such as 7.5 pm V^–1^ for lithium niobate (LiNbO_3_) and 3.1 pm V^–1^ for GaN.^16,26^ We performed more PFM tests on Bi_2_Se_3_ nanoflakes with different thicknesses. Each sample shows a linear relationship between the amplitude and applied AC voltage, as shown in Figure 4b. However, a thicker Bi_2_Se_3_ nanoflake has a smaller slope, corresponding to a smaller *d*_33_^eff^.

The thickness dependence of *d*_33_^eff^ is probably related to the work function change of the Bi_2_Se_3_ flakes. We carried out Kelvin probe force microscopy (KPFM) measurements, allowing to obtain the surface potential (*V*_sp_) of the samples, which is expressed as , where φ_S_ and φ_tip_ are the work functions of the sample and the AFM tip used, respectively, and *e* is the electronic charge. Figure 4c and e shows two Bi_2_Se_3_ nanoflakes with thicknesses of 49 and 109 nm on Au substrates (Figure S6), respectively, and Figure 4d and f shows their corresponding surface potential maps. It has been reported that the work functions of 2D materials can be affected by the adherent substrates and vary with the thickness of the 2D materials.^27^ The statistical distributions of the surface potentials show that the surface potential of the 109 nm-thick Bi_2_Se_3_ nanoflake is 94.4 mV larger than that of the 49 nm-thick nanoflake, suggesting that thicker Bi_2_Se_3_ nanoflakes have smaller work functions. Due to the work function difference between Au and Bi_2_Se_3_, there are likely interfacial effects, such as a built-in electric field near the Au/Bi_2_Se_3_ interface, that contribute to the total measured flexoelectric response.^28,29^ The decreased work functions for thicker Bi_2_Se_3_ nanoflakes may impair the interfacial effects, leading to smaller out-of-plane piezoresponses.

Similar to direct flexoelectricity, the converse-flexoelectricity-induced stress is also characterized by a fourth-rank tensor, which is expressed as
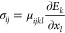
2where σ_*ij*_ is the mechanical stress tensor, μ_*ijkl*_ is the fourth-order flexoelectric tensor, and  is the electric field gradient tensor. With *ij* following the Voigt notation and *kl* following 11 → 1, 12 → 2, 13 → 3, 21 → 4, 22 → 5, 23 → 6, 31 → 7, 32 → 8, 33 → 9, the flexoelectric tensor with four indices can be transformed to the tensor with two indices, which is μ_*mn*_. Then, the flexoelectric tensor of crystals with a *R*3̅m space group is given by^30^
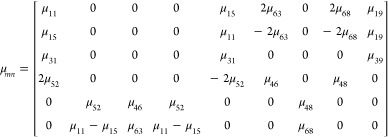
3

Since the tip is in contact with the surface of the sample and the exciting volume below the tip is rather small, the electric field inside Bi_2_Se_3_ can be considered normal to the sample surface.^16,31^ Then, the first six rows of the electric field gradient tensor can be neglected. Correspondingly in eq 3, the first six columns of the flexoelectric tensor are eliminated. Equation 2 can be simplified as
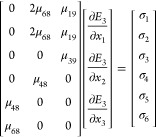
4

Equation 4 indicates that six stress components are deduced, which are
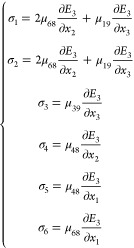
5

Among the six stress components in eq 5, σ_6_ is the in-plane shearing stress, which may lead to buckling of the AFM cantilever, contributing to the out-of-plane PFM response when σ_6_ has a component that is parallel to the AFM cantilever and pointing from the AFM tip to the cantilever end. Nevertheless, a possible induced bucking deformation by σ_6_ should be neglectable due to the insignificant value of  compared to . For instance, as shown in Figure S7, the VPFM signal amplitude from the same area of Bi_2_Se_3_ remains nearly the same before and after rotation of the sample by 180°, suggesting that there are ignorable contributions from buckling to the vertical PFM signal. Instead, σ_6_ plays a major role in the in-plane PFM signals. σ_1_ and σ_2_ are in-plane stresses, leading to in-plane contraction or expansion of the material. They may contribute to the out-of-plane surface displacements due to Poisson-like effects, which nevertheless are most likely to make a negligible contribution, as well. σ_3_, σ_4_, and σ_5_ can directly contribute to the out-of-plane PFM signal amplitude by inducing a deflection of the cantilever. Explicitly, σ_3_ is the out-of-plane stress, which is the product of  and the vertical (out-of-plane) flexoelectric coefficient, μ_39_; σ_4_ and σ_5_ are out-of-plane shearing stresses, which are generated by  and , respectively, and related to the lateral (in-plane) flexoelectric coefficient, μ_48_. Compared to ,  and  can be assumed to be neglectable. Thus, the measured *d*_33_^eff^ is considered to be mainly contributed by σ_3_, from which we can estimate μ_39_ according to eqs 5–7,
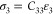
6
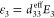
7where ε_3_ is the out-of-plane strain, and *C*_33_ is the elastic constant. Then, μ_39_ is calculated by
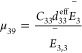
8where *E*_3_ and *E*_3,3_ are the mean values of *E*_3_ (*E*_*z*_) and  () through the thickness (*h*) of the sample, which are obtained according to eqs 9 and 10, respectively,
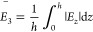
9
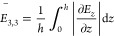
10

By adopting a value of ∼102 GPa^32^ of *C*_33_ for Bi_2_Se_3_ obtained by density functional theory (DFT) reported previously and getting the ratio of *E*_3_ to *E*_3,3_, we can calculate μ_39_ according to eq 8, which is 0.13 ± 0.01 nC m^–1^ for the 37 nm-thick sample. Similar to Bi_2_Se_3_, the out-of-plane piezoresponse of TMDCs in PFM measurements is also mainly contributed by the flexoelectric coefficient, μ_39_.^31,33^ Previous works estimated the flexoelectric coefficients of some 2D TMDCs without out-of-plane piezoelectricity using PFM. For instance, Shimada et al. measured the flexoelectric coefficient μ_39_ of MoS_2_ nanoflakes by PFM measurements, which is ∼0.27 nC m^–1^.^31^ In Yu et al.’s works, the effective flexoelectric coefficients that contribute to the out-of-plane electromechanical response of monolayers MoS_2_, MoSe_2_, and WS_2_ are estimated to be 0.065, 0.103, and 0.053 nC m^–1^, respectively.^33^ The calculated μ_39_ for Bi_2_Se_3_ nanoflakes in this work is on the same order of magnitude as the measured flexoelectric coefficients of the above 2D TMDCs.

Since odd-layered hexagonal TMDCs (2H-TMDCs) have in-plane piezoelectricity because of broken inversion symmetry, odd-layered 2H-TMDCs always have to be prepared for the building blocks of piezoelectric-effect-functionalized 2H-TMDC-based devices. The existence of flexoelectricity in centrosymmetric materials shatters the constraints that limit even-layered 2H-TMDCs in applications incorporating electromechanical coupling effects. For instance, the flexo-photovoltaic effect can be activated in a thin even-layered MoS_2_ nanosheet using strain-gradient engineering.^34^ Previous works found that the piezoelectric potential can modulate the electron transport in TIs, which paves the way to develop the TI-based piezoelectric quantum and spintronic devices.^13,35,36^ Direct flexoelectricity can generate flexoelectric potential.^37^ Inspired by the utilization of the piezoelectric potential, the flexoelectric field may also be utilized to tune the quantum states in TIs. It will be intriguing to reveal how the flexoelectric field would modulate the TI states in Bi_2_Se_3_ and other nonpiezoelectric TIs, which will be our future work. Even though the absence of piezoelectricity in Bi_2_Se_3_ hinders its application in piezotronics, the coexistence of flexoelectricity and Quantum Hall effect in it is likely to provide a method to develop flexotronics built by centrosymmetric vdW TIs.

**Figure 5:**
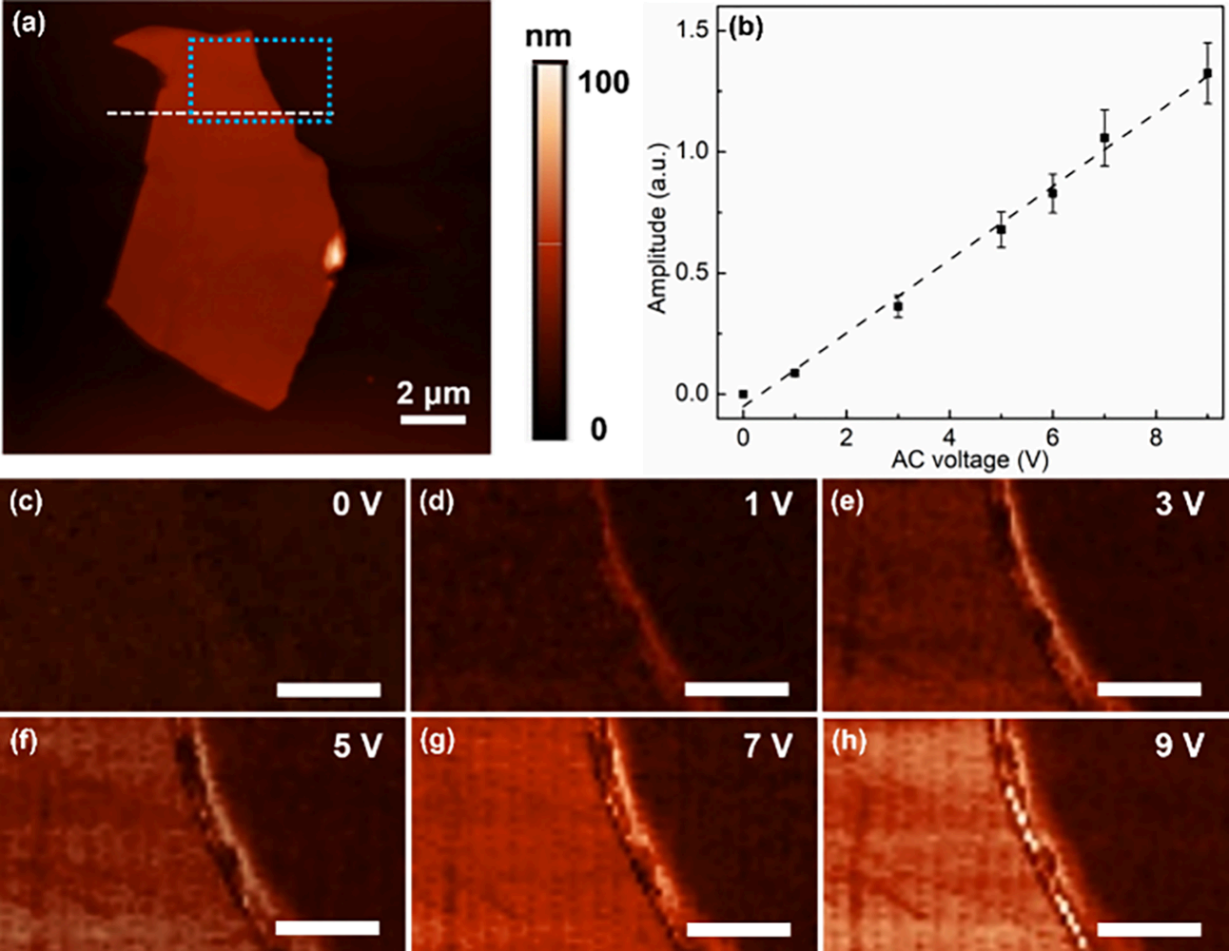
LPFM measurements of an individual Bi_2_Se_3_ nanoflake with a thickness of ∼40 nm. (a) AFM topography of the Bi_2_Se_3_ nanoflake. (b) LPFM amplitude as a function of the applied AC voltage. (c–h) In-plane PFM amplitude images of the framed region in (a) with applied AC voltages of 0, 1, 3, 5, 7, and 9 V, respectively. Scale bars: 1 μm.

As mentioned above, the stress component σ_6_ in eq 5 will cause in-plane shearing deformation. It will contribute to in-plane PFM signals when it has a component that is normal to the AFM cantilever axis. We used LPFM to study the in-plane electromechanical coupling of the Bi_2_Se_3_ nanoflakes. As shown in Figure 5c–h, the enhanced contrast of the in-plane PFM amplitude images of a Bi_2_Se_3_ nanoflake with a thickness of 40 nm (Figure S8) indicates that the amplitude increases with an increase in AC voltage increasing. Similar to the out-of-plane PFM amplitude signal, there is also a linear relationship between the in-plane amplitude and the AC voltage, as shown in Figure 5b. According to eq 5, the in-plane PFM signal originates from component μ_68_ of the flexoelectric tensor. However, it is rather difficult to calculate the value of μ_68_ unless the direction of σ_6_ is normal to the AFM cantilever axis, as shown in Figure S9.

## Conclusions

In summary, we shed light on the electromechanical properties of low-dimensional rhombohedral Bi_2_Se_3_, which is a 3D vdW TI. The Bi_2_Se_3_ nanoflake with a thickness of 37 nm has an effective out-of-plane piezoelectric coefficient of ∼0.60 pm V^–1^. The rhombohedral Bi_2_Se_3_ has a centrosymmetric crystal structure, which shows no piezoelectricity. The out-of-plane and in-plane electromechanical responses are verified to originate from the converse flexoelectricity after careful analyses. The flexoelectric coefficient mainly accounting for the measured out-of-plane effective piezoelectric coefficient is estimated to be ∼0.13 nC m^–1^, which is on the same order of magnitude as the measured effective flexoelectric coefficients of some 2D TMDCs without out-of-plane piezoelectricity. However, it is rather difficult to obtain the in-plane component of the flexoelectric tensor from the in-plane PFM measurements since the direction of the in-plane stress is always not normal to the AFM cantilever axis. The results help understand the flexoelectric effect in low-dimensional vdW TIs with centrosymmetric crystal structures. Even though vdW TIs without crystal asymmetry possess no piezoelectricity, the coexistence of flexoelectricity and Quantum Hall effect in them has implications for designing novel nano flexotronic spin devices.

## Supporting Information Available

Configuration of the PFM tests, determination of AC voltage frequency for PFM, VPFM results under DC voltages, background subtraction for calculating piezoresponse displacements, VPFM phase results, line profiles of the height, VPFM results of Bi_2_Se_3_ before and after rotation, line profile of the height, and effects of σ_6_ to piezoresponses (PDF)
