# Supplementary material for: Converse Flexoelectricity in van der Waals (vdW) Three-Dimensional Topological Insulator Nanoflakes
Source: J Phys Chem C Nanomater Interfaces. 2024 Sep 12;128(38):16265–73. doi: 10.1021/acs.jpcc.4c05690 (PMC11440597; doi:10.1021/acs.jpcc.4c05690)
Supplement: Supplementary file 1 — jp4c05690_si_001.pdf [file jp4c05690_si_001.pdf]

Supporting Information

Converse Flexoelectricity in van der Waals (vdW) Three-Dimensional Topological Insulator Nanoflakes

Qiong Liu,[a] Srivilliputtur Subbiah Nanthakumar,[a] Bin Li,[a] Teresa Cheng,[a] Florian Bittner,[c] Chenxi Ma,[d] Fei Ding,[d] Lei Zheng,[e] Bernhard Roth,[e,f] and Xiaoying Zhuang[a,b,f,] *

[a]*Chair of Computational Science and Simulation Technology, Faculty of Mathematics and Physics, Leibniz University Hannover, 30167 Hannover, Germany*

[b]*Department of Geotechnical Engineering, College of Civil Engineering, Tongji University, 200092 Shanghai, China*

[c]*Institute of Plastics and Circular Economy (IKK), Faculty of Mechanical Engineering, Leibniz University Hannover, 30823 Hannover, Germany*

[d]*Institute of Solid State Physics, Faculty of Mathematics and Physics, Leibniz University Hannover, 30167 Hannover, Germany*

[e]*Hannover Centre for Optical Technologies, Leibniz University Hannover, 30167 Hannover, Germany*

[f]*Cluster of Excellence PhoenixD (Photonics, Optics and Engineering – Innovation Across Disciplines), 30167 Hannover, Germany*

Corresponding author email: zhuang@iop.uni-hannover.de

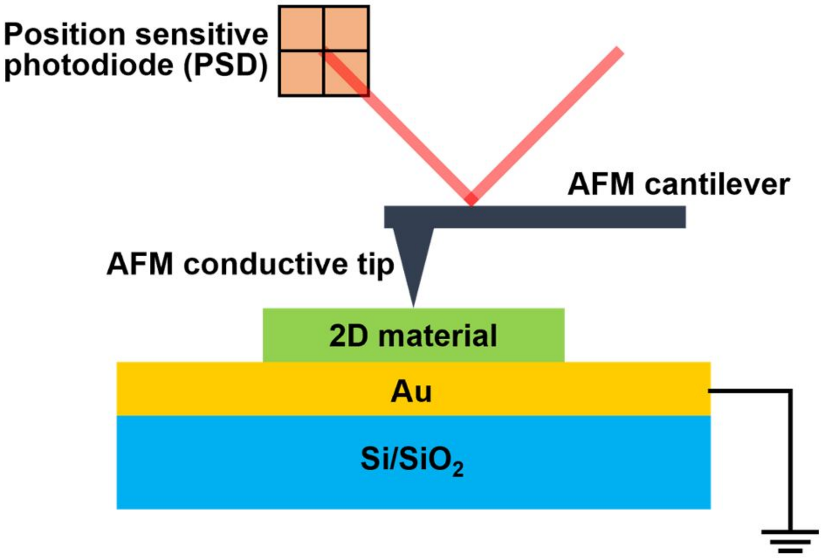

Figure S1. A schematic showing the experimental configuration of the PFM tests.

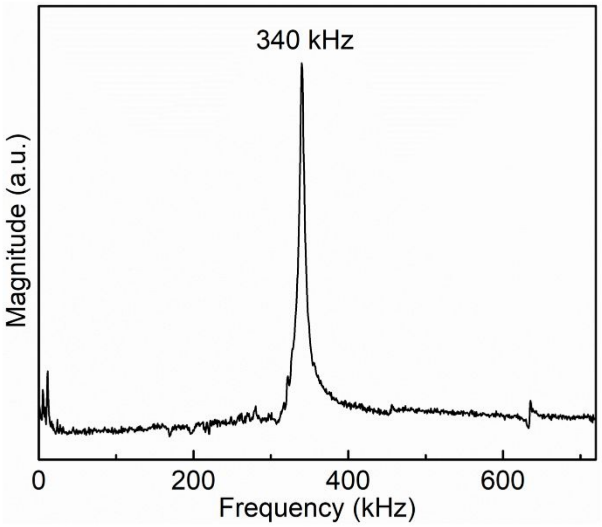

Figure S2. Determination of the frequency of the AC voltage for the PFM measurements: the piezoresponse amplitude versus AC voltage frequency measured on the Bi_2Se_3 nanoflake situated on the Au-coated Si/SiO_2 substrate. In the frequency regime below 30 kHz and between 150 to 300 kHz, the amplitude signal shows a fluctuation. The contact resonance frequency of the AFM cantilever is 340 kHz. In the PFM measurements of the samples, the frequency of 60 kHz for the AC voltage is chosen, which is far from the contact resonance frequency and within the regime for steady amplitude signal.

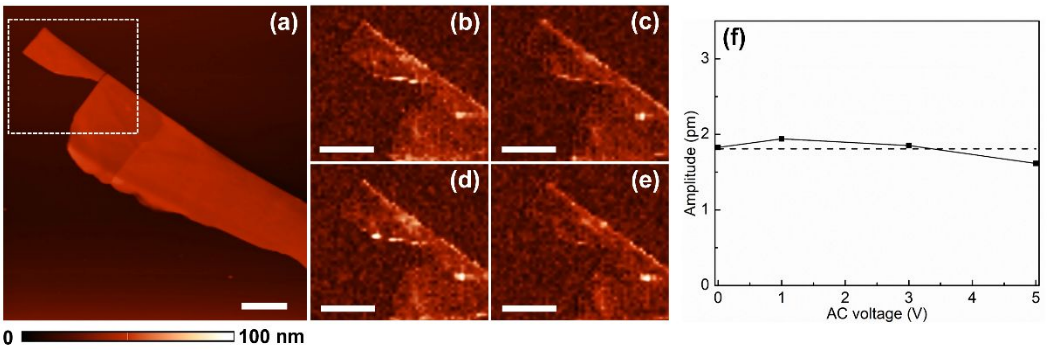

Figure S3. VPFM measurements on an individual Bi_2Se_3 nanoflake with different DC voltage at a constant AC voltage of 8V. (a) AFM topography, (b-e) PFM amplitude images of the nanoflake with DC voltages of 0, 1, 3, and 5 V, respectively. Scale bars, 2 μm. (f) Values of PFM amplitude. The dash line represents the mean value.

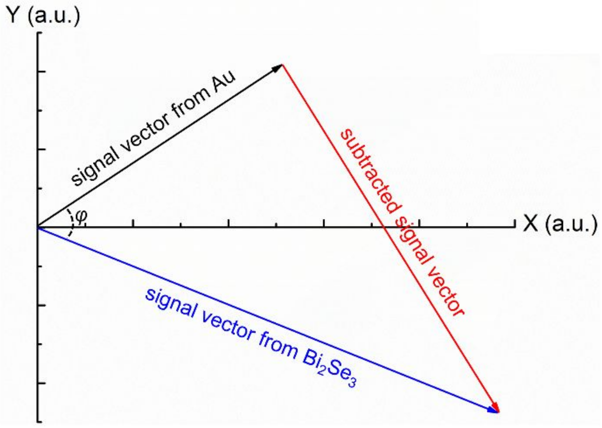

Figure S4. A schematic showing the background subtraction method for obtaining the actual PFM amplitude signal of samples. X and Y represent the *x*- and *y*-components of the amplitude, respectively. With the amplitude and phase signals acquired, a vector can be drawn. The black and blue arrows are signal vectors from Au and Bi_2Se_3, respectively, with an angle, φ, in between; by using the blue arrow to subtract the black arrow, a background subtracted signal vector can be obtained, whose magnitude is the actual amplitude originating from Bi_2Se_3 and can be calculated according to the law of cosines. When the phase signals from the Au and the sample are close, φ is pretty small. In this case, the actual amplitude signal from Bi_2Se_3 can be considered to be the difference of the magnitude of the signal vector from Bi_2Se_3 and the signal vector from Au.

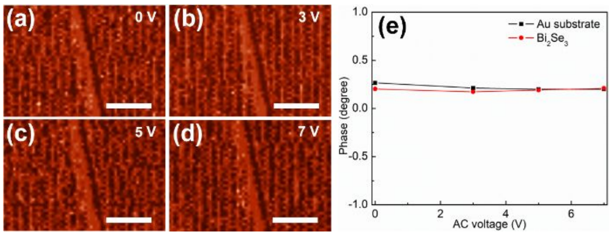

Figure S5. VPFM phase results of the Bi_2Se_3 nanoflake in Figure 3. (a-d) PFM phase images and (e) comparison of the phase values from Bi_2Se_3 and Au measured at AC voltages of (c) 0, (d) 3, (e) 5, and (f) 7 V, respectively. Scale bars, 2 μm.

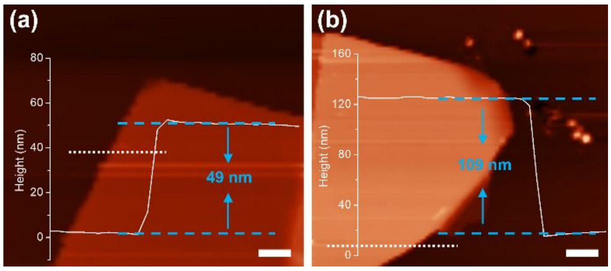

Figure S6. (a) AFM topography image of a Bi_2Se_3 nanoflake with a thickness of 49 nm. Inset is the height profile along the dashed line. (b) AFM topography image of a Bi_2Se_3 nanoflake with a thickness of 109 nm. Inset is the height profile along the dashed line. Scale bars, 5 μm.

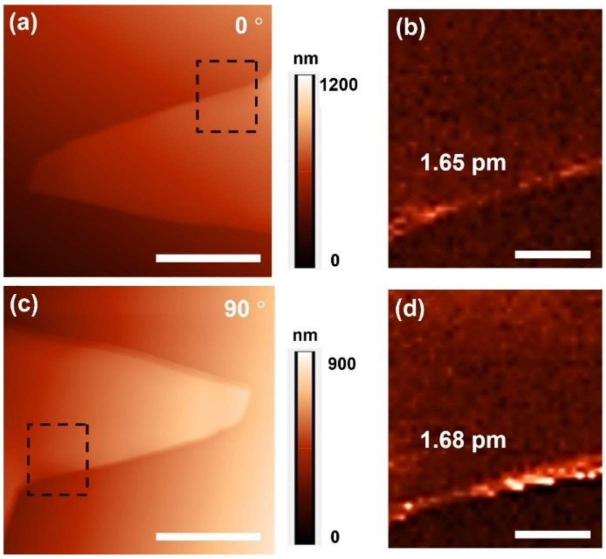

Figure S7. VPFM measurements of a Bi_2Se_3 nanoflake for ruling out the AFM cantilever buckling. (a,c) AFM topography images, and (b,d) PFM amplitude images of the nanoflake before and after rotation by 180°. The deformation amplitudes are 1.65 and 1.68 pm, respectively. Scale bars, 5 μm in a and c, 1 μm in b and d.

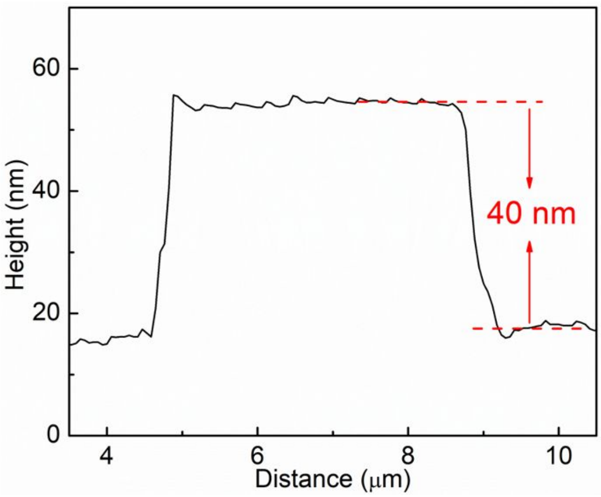

Figure S8. Line profile of the height along the dashed line in Figure 5a.

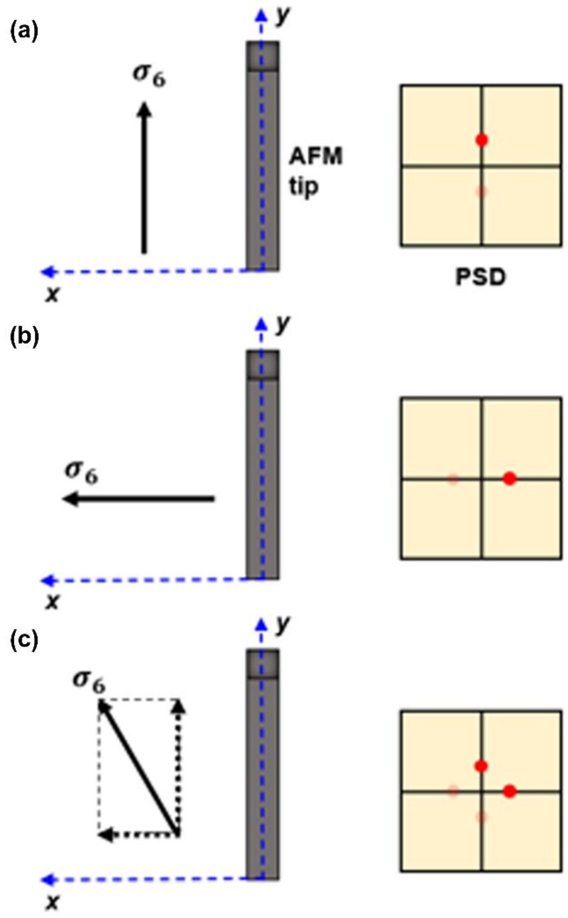

Figure S9. Schematic showing the effects of the direction of σ_6 to the VPFM and LPFM responses. (a) When σ_6 is parallel to the AFM tip, only out-of-plane piezoresponse is likely to be generated. (b) When σ_6 is vertical to the AFM tip, only in-plane piezoresponse is likely to be generated. (c) When σ_6 is neither parallel nor vertical to the AFM tip, both out-of-plane and in-plane piezoresponse may be generated.
